# The Teflon hypothesis

**DOI:** 10.1093/braincomms/fcad203

**Published:** 2023-07-13

**Authors:** Rudy J Castellani, George Perry

**Affiliations:** Department of Pathology, Northwestern University Feinberg School of Medicine, Chicago, IL 60611, USA; Department of Neuroscience, Developmental and Regenerative Biology, The University of Texas at San Antonio, San Antonio, TX 78249, USA

## Abstract

This scientific commentary refers to ‘Key questions for the evaluation of anti-amyloid immunotherapies for Alzheimer's disease', by Liu *et al*. (https://doi.org/10.1093/braincomms/fcad175).

**This scientific commentary refers to ‘Key questions for the evaluation of anti-amyloid immunotherapies for Alzheimer's disease', by Liu** *et al* **. (https://doi.org/10.1093/braincomms/fcad175).**

‘Target engagement’ from active immunization and apparent clearing of amyloid-β plaques have long been accepted outcomes of the AN-1792 clinical trial from two decades ago.^1^ The 6% risk of meningoencephalitis was intolerable to government agencies, so research stopped. Today, target engagement and apparent clearing of amyloid-β plaques are accepted as outcomes in trials of passive immunotherapy, with neurovascular toxicity (amyloid-related imaging abnormalities, or ARIA) as high as 43% in certain subgroups,^2^ and an anecdotal account of iatrogenic death.^3,4^ Yet this is tolerable to government agencies, so much so that they both approve and pay for the passive immunotherapy. So what changed? Ostensibly, outcome data suggest a reduced rate of cognitive deterioration following complex analysis of largely opaque information and the lately codified emphasis on ‘improvement’ via biomarkers, both insensible to the individual patient.

Recently in *Brain Communications*, Liu *et al.*^5^ review the topic of amyloid-targeting immunotherapy and raise three important questions related to the claims of efficacy and safety. The simple fact that these questions arise might be cause for concern. The authors conclude, ‘While amyloid-targeting immunotherapies may be promising disease-modifying Alzheimer's disease treatments, rigorous and unbiased assessment of clinical trial data is critical to regulatory decision-making and subsequently determining their provision and utility in routine clinical practice.’ By inference, the clinical trial data presented to the public, in the view of the authors, are biased and inadequately rigorous. The authors further highlight concerns about brain shrinkage, concerns about patient samples lacking the ethnicity profile for the broader community and advocacy-oriented language that inflates the perception of benefits and deflates the risk. In the end, it appears that clinical efficacy is unproven, human diversity is not adequately accounted for, and claims of safety require minimizing brain toxicity while rationalizing iatrogenic death.

The amyloid cascade hypothesis has thus become a powerful narrative that is about to infiltrate Alzheimer's disease patients on a broad scale, some of whom may not survive toxic side effects of the new therapy. Once the asymmetry between toxicity and benefit is fully appreciated, however, one can then anticipate an expanding discussion of Alzheimer's disease ‘polypathology’, a trivial oversimplification more and more invoked to rationalize modest or negative clinical trial results. Amyloid-β will then be relegated to a smaller and smaller piece of a pie that is Alzheimer’ disease, as we await constructs to address such phenomena as phospho-tau, alpha-synuclein, TDP-43, neurovascular disease and whatever pathophysiology amenable to small molecule and biologic targeting that might emerge. Anti-amyloid-β treatment, though toxic and of questionable efficacy, will continue nonetheless, being the cornerstone of the Teflon hypothesis known as the amyloid cascade.
